# **Radiotherapy** **dose** **may** **not** **affect** **prognosis** **in** **ESCC** **patients** **receiving** **first-line** **chemoradiotherapy**-**immunotherapy**: **A** **multicenter** **retrospective** **study**

**DOI:** 10.1007/s00262-025-04261-3

**Published:** 2026-01-27

**Authors:** Jingyuan Wen, Xinyi Liu, Yuanji Xu, Zhongmei Lin, Min Hou, Yan Gui, Jianzhong Cao, Qing Hou, Jiahua Lv, Lulu Wang, Wei Zhou, Zhimin Zeng, Wenbin Shen

**Affiliations:** 1 Department of Radiation Oncology, The Fourth Hospital of Hebei Medical University, Shijiazhuang, 050035 China; 2 Department of Radiation Oncology, Clinical Oncology School of Fujian Medical University, Fujian Cancer Hospital, Fuzhou, 350014 China; 3 Department of Oncology, Affiliated Hospital of North Sichuan Medical College, Nanchong, 637000 China; 4 Department of Radiation Oncology, Shanxi Province Cancer Hospital, Cancer Hospital Affiliated to Shanxi Medical University, Taiyuan, 030013 China; 5 Department of Radiotherapy, Sichuan Clinical Research Center for Cancer, Sichuan Hospital Cancer & Institute, Sichuan Cancer Center, University of Electronic Science and Technology of China, Chengdu, 610040 China; 6 Department of Radiation Oncology, Chongqing University Cancer Hospital, Chongqing, 400030 China; 7 Department of Oncology, The Second Affiliated Hospital of Nanchang University, Nanchang, 330008 China

**Keywords:** Esophageal squamous cell carcinoma, Radiotherapy doses, Immunotherapy, Chemotherapy, Real-world data

## Abstract

**Background:**

Combined immunotherapy based on radiotherapy and chemotherapy is increasingly widely applied in clinical practice for patients with non-surgical treatment of esophageal cancer. However, radiotherapy doses in triple combination therapy have not received much attention. Therefore, a retrospective, non-interventional, real-world study of patients with esophageal squamous cell carcinoma (ESCC) was conducted. The primary objective was to assess whether radiotherapy dose is a determining factor in the prognosis of ESCC patients in a triple therapy.

**Methods:**

A total of 1283 ESCC patients receiving triple therapy were collected from 7 cancer centers in China between January 2019 and December 2022. Among them, 299 ESCC patients receiving the first-line triple therapy were eligible for enrollment. Due to different radiotherapy doses, patients were classified into a high-dose (HD) group at 60Gy and a low-dose (LD) group at 50.4Gy. Propensity Score Matching (PSM) analysis was conducted to compare and analyze differences in outcomes, toxicity, and failure patterns between the two groups. Further subgroup analysis was performed to identify the individual population.

**Results:**

Of the 299 ESCC patients eligible for enrollment, 198 (66.2%) were in the HD group and 101 (33.8%) were in the LD group. After PSM, there were 93 patients in each group. The median follow-up time was 25.5 months (95CI: 18.6–32.4). The median overall survival (mOS) and median progression-free survival (mPFS) in the LD group were 31.3 months (95%CI: 16.5–46.2) and 17.0 months (95%CI: 15.1–19.0), respectively. The mOS and mPFS in the HD group were 28.5 months (95%CI: 16.1–40.9) and 20.6 months (95%CI: 13.5–27.8), respectively. There was no statistically significant difference between the HD group and the LD group (X^2^ = 0.057, 0.974, *P* = 0.811, 0.324). The disease control rates of LD and HD groups were 89.2% and 90.3% respectively, and the difference was not statistically significant (X^2^ = 0.059, *P* = 0.809). In the LD group, 35 cases (37.6%) had distant metastasis and 21 cases (22.6%) had local recurrence. In the HD group, 32 cases (34.4%) had distant metastasis and 14 cases (15.1%) had local recurrence. There were no statistically significant differences between the HD group and the LD group (X^2^ = 1.725, 0.210, *P* = 0.189, 0.647).

**Conclusion:**

Multicenter data from China showed that higher radiotherapy doses provide no survival benefit for ESCC patients receiving first-line triple therapy.

## Introduction

Esophageal cancer is one of the most frequent malignant tumors and the sixth leading cause of cancer-related death worldwide [1]. ESCC is the dominant histological subtype of esophageal cancer [2]. In China, ESCC patients represent more than half of the global total, with about 40% of patients initially diagnosed with advanced disease [1, 2]. Chemotherapy based on platinum was once the standard first-line treatment, with less than 13 months of the median overall survival [2, 3]. In recent years, immunotherapy has changed the therapeutic landscape for advanced ESCC. The phase 3 randomized clinical trials show that anti-PD-1 immunosuppressant combined chemotherapy has better survival than chemotherapy alone in the first-line treatment for advanced ESCC. Despite PD-L1 expression status, the median overall survival (OS) is 13.2–17.2 months in the overall population [4, 5]. Thus, chemotherapy combined with immunotherapy is established as the new standard first-line treatment for advanced ESCC.

Radiotherapy (RT) is often used to relieve the local symptoms of advanced ESCC, such as dysphagia, pain, and difficult breathing [6]. Multiple studies have shown that RT can improve local and systemic anti-tumor immune responses through multiple mechanisms [7, 8]. Additionally, PD-1 inhibitors can promote vascular normalization to enhance radiosensitivity and overcome hypoxia [9, 10]. Compared to RT alone or anti-PD-1 monotherapy, the synergistic effect of radiotherapy combined with immunotherapy produces significant survival benefits in locally advanced ESCC [11, 12]. At present, chemoradiotherapy combined with immunotherapy shows better survival in locally advanced esophageal cancer and has gradually become mainstream [13–15].

However, radiotherapy dose is a controversial issue in clinical practice. The Radiation Therapy Oncology Group (RTOG) 85-01 study [16, 17] showed that a 50 Gy dose of radiotherapy concurrent with chemotherapy is the standard treatment for patients with localized esophageal cancer. However, the local failure rate after radical radiotherapy is high (47%), which has been confirmed in several other large radical chemoradiotherapy (dCRT) studies [18, 19]. Compared to neck, head and lung cancers, the dose of 50Gy used in the dCRT regimen for esophageal cancer is relatively low, with a poor local control rate [20, 21]. To improve local control, the use of high-dose radiotherapy (64.8 Gy/1.8 Gy) was compared with the standard dose (50.4 Gy/1.8 Gy) combined with chemotherapy using the RTOG 94-05 randomized trial [18]. The results showed no significant differences in local recurrence (52% vs 56%) or 2-year overall survival (31% vs 40%). The ARTDECO randomized study [22], which increased the dose from50.4Gy to 61.6Gy, also failed to enhance local tumor control and survival. Thus, the dose of 50.4 Gy is still considered the standard dose. However, several non-randomized retrospective studies have shown that survival can be improved by increasing local radiotherapy doses [23–26]. Given the different biological characteristics between ESCC and esophageal adenocarcinoma, currently, Chinese clinicians generally use a dose of 60 Gy with modern radiological techniques, as they believe that 50–50.4 Gy is insufficient for ESCC [26]. Currently, the relevant research on the best radiotherapy dose for ESCC patients receiving the first-line combination therapy is still rare. Therefore, this study conducted a cohort study based on multi-center data from China to compare the clinical effects of 60 Gy and 50.4 Gy radiotherapy doses in ESCC patients receiving first-line chemoradiotherapy combined with immunotherapy.

## Materials and methods

### Study design

This study (ClinicalTrials.gov NCT06478355) was a multicenter, retrospective, non-interventional real-world research conducted in seven major cancer centers in China (The Fourth Hospital of Hebei Medical University, Clinical Oncology School of Fujian Medical University, Affiliated Hospital of North Sichuan Medical College, Cancer Hospital Affiliated to Shanxi Medical University, Sichuan Cancer Center, Chongqing University Cancer Hospital, The Second Affiliated Hospital of Nanchang University), including first-line patients with advanced ESCC receiving chemoradiotherapy combined with immunotherapy. The medical records of ESCC patients receiving immunotherapy in seven hospitals were screened. Inclusion criteria included: (i) age > 18 years old; (ii) pathological evidence of ESCC; (iii) the clinical stage was cTanyNanyM0, as indicated by Tumor Node Metastasis (TNM) staging criteria (8th edition); (iv) receiving chemoradiotherapy combined with immunotherapy between January 2019 and December 2022, both for the first time; (v) receiving a radiotherapy dose of 50.4 Gy or 60 Gy; (vi) no previous anti-cancer treatment. Exclusion criteria included: (i) a history of other malignancies; (ii) autoimmune disease or serious infection; (iii) multicenter primary tumors of the esophagus. This study was approved by the Ethics Committee of the Fourth Hospital of Hebei Medical University (approval number: 2024KY167). As this study is a retrospective one, informed consent was waived. The study complied with the Declaration of Helsinki.

### Procedures

#### Immunotherapy

All patients received carrilizumab, 200 mg/ time intravenously, once every 3 weeks. The number of use cycles ranged from 1 to 33, with a median of 4.

#### Chemotherapy

All patients received chemotherapy drugs recommended by the guidelines or expert consensus. Among them, there were 44 cases (14.7%) of platinum combined with paclitaxel and 255 cases (85.3%) of platinum combined with albumin-bound paclitaxel. The chemotherapy cycles ranged from 2 to 6, with a median of 4.

#### Radiotherapy

All patients received radiotherapy after 1–2 cycles of chemotherapy combined with immunotherapy. All patients received radiotherapy for esophageal lesions and/or metastatic lymph nodes using intensity modulated radiation therapy (IMRT) with a high energy (6MV) linear accelerator, volume modulated radiation therapy (VMAT), or the spiral TOMO system. The gross target area (GTV) contained the tumor bed of esophageal lesions. The clinical target area (CTV) was 2–3 cm at the upper and lower ends and 0.5–1.0 cm at the left and right sides. On this basis, the planned target area (PTV) was uniformly extended by 0.5–0.8 cm. Enlarged lymph nodes (the shortest diameter of mediastinal and supraclavicular lymph nodes ≥ 10 mm, and the shortest diameter of parafoesophageal and abdominal lymph nodes ≥ 0.5 cm) were characterized as GTV-nd. On this basis, uniformly expanded outward by 0.5–0.8 cm was characterized as PTV-nd. PTV/PTV-nd received a dose of 50.4 Gy/28 times, 1.8 Gy/time or 60 Gy/30 times, 2.0 Gy/time of conventional segmentation irradiation. Peripheral dose-limiting organs and tissues should be avoided when mapping the target area.

### Follow-up

Adverse events related to treatment were assessed every 3 weeks during subsequent anti-PD-1 consolidation therapy and weekly during chemoradiotherapy. After treatment, patients were followed regularly every 1–3 months for the first two years, every 6 months for the next three years, and annually. The main content included routine physical examination, blood examination, chest/abdomen CT scan, esophagography, PET/CT examination when necessary, electronic gastroscopy for patients with suspected esophageal recurrence, and needle aspiration biopsy for pathology when there is superficial lymph node enlargement. Treatment after disease progression was determined by consultation between the treating physician and the patient.

### Outcomes

The primary endpoints consisted of progression-free survival (PFS) and OS. PFS is defined as the time from the start of treatment to the last follow-up or the onset of disease progression. OS is defined as the time from the start of treatment to the last follow-up or death from any cause. According to the 1.1 version of response evaluation criteria in solid tumors (RECIST1.1), partial response (PR) refers to a reduction of ≥ 30% in the sum of the maximum diameter of the tumor target lesion for at least 4 weeks. Complete response (CR) means that all the tumor target lesions disappear without new lesions and the tumor markers are normal for at least 4 weeks. Stable disease (SD) refers to no increase to progression disease (PD) or no decrease to PR in the sum of the maximum diameter of the tumor target. PD refers to the appearance of new lesions or growth of at least 20% in the sum of the maximum diameter of the tumor target lesion. Disease control rate (DCR) represents the ratio of patients with tumor lesions achieving CR, PR and SD. The objective response rate (ORR) represents the ratio of patients with tumor lesions achieving CR and PR.

### Statistical analysis

Differences in the distribution of factors among the groups were evaluated using the Pearson χ^2^ test. OS and PFS were estimated using the Kaplan–Meier method. Inter-group differences were assessed using the log-rank test. Prognostic analysis of OS and PFS was performed by univariate and multifactor Cox proportional risk regression models (*P* < 0.05). Factors between different radiotherapy dose cohorts were balanced using PSM. Matching variables included sex, age, smoking, alcohol consumption, cTNM stage, cN stage, cT stage, tumor location, Eastern Cooperative Oncology Group Performance Status (ECOG PS) score, chemotherapy cycle, and immunotherapy cycle. The effects of different factors on survival time were evaluated using unifactor COX regression analysis. Subgroup analysis forest plots were drawn using R language forest plots. Pearson χ^2^ test or continuous correction χ^2^ test was used to compare failure modes and adverse reactions. R version 4.4.2 and SPSS version 26.0 were used for all statistical analyses, with statistical significance at the two-tailed *P* < 0.05.

## Results

### Patient characteristics

**Fig. 1:**
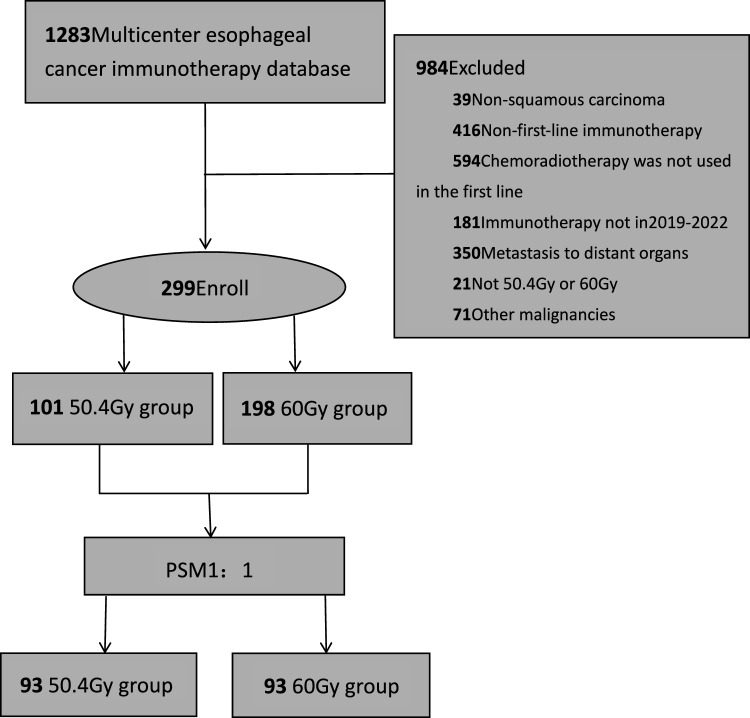
Flow chart of patient inclusion

A total of 1283 patients with esophageal cancer from seven cancer centers in China were screened using immunotherapy. Then, 299 patients were enrolled after screening for inclusion and exclusion criteria (Fig. 1). Due to different radiotherapy doses, patients were classified into the LD group receiving 50.4 Gy and the HD group receiving 60 Gy, with 101 patients (33.8%) and 198 patients (66.2%), respectively. The composition ratio analysis of general clinicopathological data showed statistically significant differences in tumor location and N stage between the two groups (*P* = 0.026, 0.006). PSM analysis was conducted in the two groups (1:1), with 93 patients in each group after PSM. See Table 1 for details.

**Table 1:** Baseline characteristics before and after PSM

Characteristics	Subgroup	RT dose before PSM(n = 299)			RT dose after PSM(n = 186)		
		50.4 Gy(101)	60 Gy(198)	*P*	50.4 Gy(93)	60Gyy(93)	*P*
Gender	male	72(71.3)	153(77.3)	0.257	66(71.0)	71(76.3)	0.439
	female	29(28.7)	45(22.7)		27(29.0)	22(23.7)	
Age	< 70	71(70.3)	147(74.2)	0.468	65(769.9)	63(67.7)	0.647
	≥ 70	30(29.7)	51(25.8)		28(30.1)	30(32.3)	
ECOG	0–1	87(86.1)	181(91.4)	0.157	80(86.0)	84(90.3)	0.270
	2–3	14(13.9)	17(8.6)		13(14.0)	9(9.7)	
Smoking	No	65(64.4)	107(54.0)	0.088	58(62.4)	58(62.4)	0.107
	Yes	36(35.6)	91(46.0)		35(37.6)	35(37.6)	
Drinking	No	64(63.4)	122(61.6)	0.768	58(62.4)	61(65.6)	0.317
	Yes	37(36.6)	76(38.4)		35(37.6)	32(34.4)	
Tumor location	Cervical	6(5.9)	20(10.1)	0.026	6(6.5)	6(6.5)	0.099
	Upper thoracic	24(23.8)	67(33.8)		22(23.7)	22(23.7)	
	Middle thoracic	49(48.5)	78(39.4)		46(49.5)	42(45.2)	
	Lower thoracic	22(21.8)	33(16.7)		19(20.4)	23(24.7)	
cT	T1-2	11(10.9)	25(12.6)	0.155	11(11.8)	17(18.3)	0.650
	T3	74(73.3)	118(59.6)		72(77.4)	62(66.7)	
	T4	16(15.8)	55(27.8)		10(10.8)	14(15.1)	
cN	N0	6(5.9)	24(12.1)	0.006	6(6.5)	7(7.5)	0.006
	N1	28(27.7)	65(32.8)		28(30.1)	18(19.4)	
	N2	47(46.5)	91(46.0)		45(48.4)	58(62.4)	
	N3	20(19.8)	18(9.1)		14(15.1)	10(10.8)	
cTNM	I-II	10(9.9)	27(13.6)	0.823	10(10.8)	15(16.1)	0.124
	III	63(62.4)	100(50.5)		61(65.6)	55(59.1)	
	Ⅳa	28(27.7)	71(35.9)		22(23.7)	23(24.7)	
Chemotherapy cycle	< 4	29(28.7)	70(35.4)	0.248	27(29.0)	30(32.3)	0.575
	≥ 4	72(71.3)	128(64.6)		66(71.0)	63(67.7)	
Immunotherapy cycle	< 3	20(19.8)	60(30.3)	0.052	20(21.5)	26(28.0)	0.125
	≥ 3	81(80.2)	138(69.7)		73(78.5)	67(72.0)	

### Single-factor and multi-factor analysis and comparison of failure modes before PSM

For all patients, the median follow-up time before PSM was 32.6 months (95%CI: 29.8–35.3). The OS and PFS rates at 1, 2 and 3 years were 79.7%, 59.7%, 50.7% and 66.7%, 43.5%, and 37.0%, respectively. The mOS and mPFS time were 37.8 (95%CI: 29.0–46.6) and 19.2 (95%CI: 15.3–23.1) months, respectively.

**Fig. 2:**
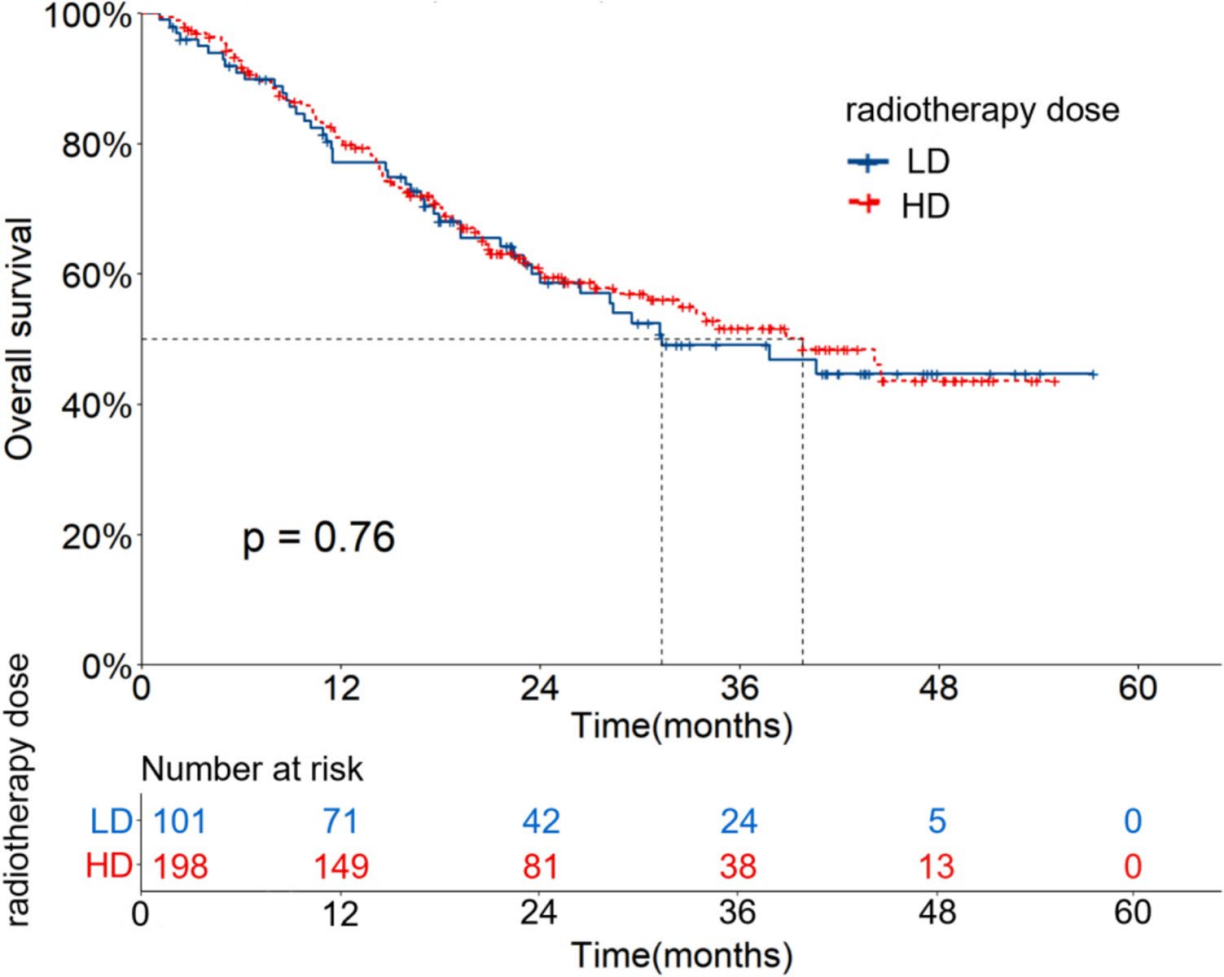
OS curves of patients with different radiotherapy doses before PSM

**Fig. 3:**
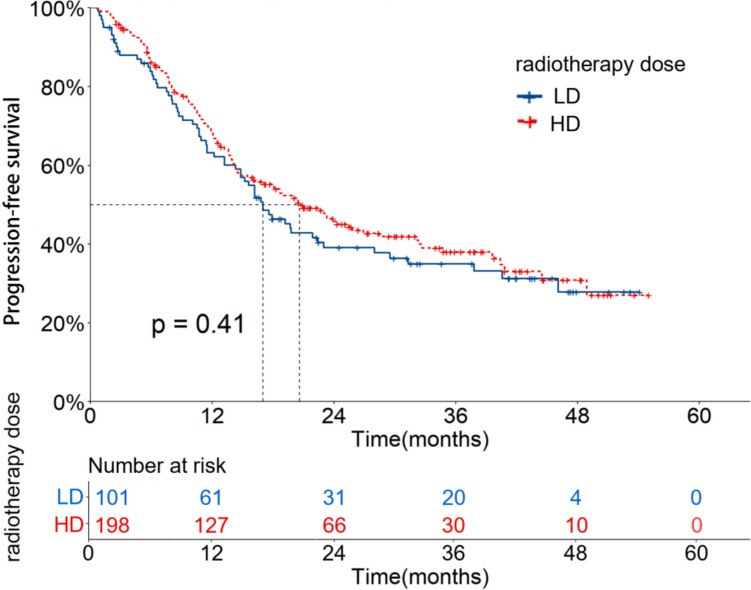
PFS curves of patients with different radiotherapy doses before PSM

In the LD group, the OS and PFS rates at 1, 2 and 3 years were 77.1%, 58.6%, 49.1% and 63.2%, 39.1%, 35.0%, respectively. The mOS and mPFS were 31.3 (95%CI: 18.5–44.2) and 17.0 (95%CI: 13.1–20.8) months, respectively. In the HD group, the OS and PFS rates at 1, 2 and 3 years were 81.0%, 60.2%, 51.7% and 69.4%, 45.8%, 38.0%, respectively. The mOS and mPFS were 39.8 (95%CI: 29.9–49.7) and 20.6 (95%CI: 15.0–26.3) months, respectively. There were no significant differences in OS and PFS between the two groups (X^2^ = 0.095, 0.670, *P* = 0.758, 0.413). The OS and PFS curves are shown in Figs. 2 and 3.

According to univariate analysis, age, gender, ECOG score, cT stage and number of immunotherapy cycles were significant factors affecting OS, Hazard Ratio (HR) = 1.62, 1.47, 2.11, 2.46, 0.64, *P* = 0.031, 0.039, 0.001, 0.012, 0.017. Age, gender, ECOG score and number of immunotherapy cycles were significant factors affecting PFS (HR = 1.79, 1.64, 2.41, 0.55, *P* = 0.045, 0.034, 0.002, 0.015). Cox multivariate analysis showed that patients with ECOG PS score 0–1, cT1-2, and immunotherapy cycle number ≥ 3 had better OS (HR = 2.17, 2.44, 0.59, *P* = 0.001, 0.014, 0.007). Women and patients with ECOG PS scores 0–1 had better PFS (HR = 1.68, 1.68, *P* = 0.007, 0.020). See Tables 2 and 3 for details.

**Table 2:** Single factor and multifactor analysis of OS before and after PSM

Characteristics	Subgroup	Before PSM(n = 299)				After PSM(n = 186)			
		Univariate analysis		Multivariate analysis		Univariate analysis		Multivariate analysis	
		HR(95%CI)	*P*	HR(95%CI)	*P*	HR(95%CI)	*P*	HR(95%CI)	*P*
Gender	female								
	male	1.62 (1.05–2.51)	0.031	1.43(0.92–2.23)	0.112	1.79 (1.01–3.15)	0.045	1.37(0.76–2.47)	0.289
Age	<70								
	≥70	1.47 (1.02–2.13)	0.039	1.38(0.95–2.01)	0.094	1.64 (1.04–2.6)	0.034	1.7(1.06–2.71)	0.026
ECOG	0–1								
	2–3	2.11 (1.34–3.31)	0.001	2.17(1.37–3.44)	0.001	2.41 (1.37–4.26)	0.002	2.48(1.39–4.42)	0.002
Smoking	No								
	Yes	1.14 (0.81–1.62)	0.453			1.44 (0.91–2.28)	0.124		
Drinking	No								
	Yes	1.17 (0.82–1.66)	0.392			1.29 (0.8–2.06)	0.294		
Tumor location	Cervical								
	Upper thoracic	0.56 (0.3–1.05)	0.069			0.69 (0.25–1.92)	0.479		
	Middle thoracic	0.75 (0.42–1.33)	0.324			1.14 (0.45–2.9)	0.777		
	Lower thoracic	0.62 (0.31–1.21)	0.161			0.89 (0.32–2.46)	0.824		
cT	T1-2								
	T3	1.63 (0.84–3.14)	0.146	1.71(0.87–3.34)	0.117	1.44 (0.69–3.03)	0.332		
	T4	2.46 (1.22–4.94)	0.012	2.44(1.2–4.97)	0.014	1.7 (0.68–4.24)	0.253		
cN	N0								
	N1	1.49 (0.72–3.09)	0.280			0.54 (0.21–1.43)	0.216		
	N2	1.71 (0.85–3.43)	0.133			1 (0.43–2.33)	0.995		
	N3	1.46 (0.65–3.31)	0.363			0.46 (0.15–1.42)	0.178		
cTNM	I-II								
	III	1.59 (0.82–3.07)	0.172			1.00 (0.51–1.98)	0.998		
	Ⅳa	1.92 (0.97–3.79)	0.062			0.74(0.33–1.66)	0.471		
Chemotherapy cycle	<4								
	≥4	1.21 (0.83–1.76)	0.332			1.41 (0.84–2.36)	0.193		
Immunotherapy cycle	<3								
	≥3	0.64 (0.44–0.92)	0.017	0.59(0.4–0.86)	0.007	0.55 (0.34–0.89)	0.015	0.54(0.33–0.88)	0.014
Radiotherapy dose	50.4 Gy								
	60 Gy	0.94 (0.66–1.36)	0.756			1.06 (0.68–1.67)	0.791

**Table 3:** Single factor and multifactor analysis of PFS before and after PSM

Characteristics	Subgrou*p*	Before PSM(n = 299)				After PSM(n = 186)			
		Univariate analysis		Multivariate analysis		Univariate analysis		Multivariate analysis	
		HR(95%CI)	*P*	HR(95%CI)	*P*	HR(95%CI)	*P*	HR(95%CI)	*P*
Gender	female								
	male	1.79 (1.01–3.15)	0.045	1.68(1.15–2.44)	0.007	1.51 (0.94–2.43)	0.086		
Age	< 70								
	≥ 70	1.64 (1.04–2.6)	0.034	1.06(0.76–1.47)	0.739	1.27 (0.85–1.9)	0.239		
ECOG	0–1								
	2–3	2.41 (1.37–4.26)	0.002	1.68(1.08–2.6)	0.020	2.36 (1.37–4.04)	0.002	2.36(1.37–4.08)	0.002
Smoking	No								
	Yes	1.44 (0.91–2.28)	0.124			1.45 (0.98–2.15)	0.063		
Drinking	No								
	Yes	1.29 (0.8–2.06)	0.294			1.45 (0.97–2.16)	0.068		
Tumor location	Cervical								
	Upper thoracic	0.69 (0.25–1.92)	0.479			0.41 (0.18–0.94)	0.036	0.4(0.18–0.92)	0.030
	Middle thoracic	1.14 (0.45–2.9)	0.777			0.63 (0.3–1.33)	0.223	0.59(0.28–1.26)	0.174
	Lower thoracic	0.89 (0.32–2.46)	0.824			0.61 (0.27–1.37)	0.231	0.55(0.24–1.23)	0.146
cT	T1-2								
	T3	1.44 (0.69–3.03)	0.332			0.83 (0.48–1.44)	0.510		
	T4	1.7 (0.68–4.24)	0.253			1.33 (0.67–2.64)	0.414		
cN	N0								
	N1	0.54 (0.21–1.43)	0.216			0.84 (0.36–1.99)	0.696		
	N2	1 (0.43–2.33)	0.995			1.18 (0.54–2.58)	0.680		
	N3	0.46 (0.15–1.42)	0.178			0.7 (0.27–1.85)	0.474		
cTNM	I-II								
	III	0.92 (0.57–1.49)	0.740			0.91 (0.51–1.62)	0.745		
	Ⅳ	1.16(0.7–1.92)	0.559			0.86 (0.45–1.67)	0.661		
Chemotherapy cycle	< 4								
	≥ 4	1.41 (0.84–2.36)	0.193			1.35 (0.86–2.09)	0.188		
Immunotherapy cycle	< 3								
	≥ 3	0.55 (0.34–0.89)	0.015	0.89(0.63–1.25)	0.512	0.79 (0.51–1.23)	0.303		
Radiotherapy dose	50.4 Gy								
	60 Gy	0.88 (0.65–1.19)	0.409			0.83 (0.56–1.22)	0.339		

After treatment, the efficacy evaluation of the LD group was PD in 13 cases (12.9%), SD in 28 cases (27.7%), PR in 29 cases (28.7%) and CR in 31 cases (30.7%). The HD group had 19 cases (9.6%) of PD, 57 cases (28.8%) of SD, 59 cases (29.8%) of PR, and 63 cases (31.8%) of CR. The two groups had DCR values of 87.1% and 90.4%, respectively, with no statistical significance (X^2^ = 0.751, *P* = 0.386). The two groups had the ORR value of 59.4% and 61.6%, respectively, with no statistical significance (X^2^ = 0.137, *P* = 0.711).

As of the follow-up date, 64 (60.2%) patients in the LD group had disease progression, of which 25 (24.8%) had local recurrence and 39 (38.6%) had distant metastasis. Besides, 113 patients (49.5%) in the HD group had disease progression, of which 36 patients (18.2%) had local recurrence and 77 patients (38.9%) had distant metastasis. Neither local recurrence nor distant metastasis rates were statistically significant between the two groups (X^2^ = 1.778, 0.002, *P* = 0.182, 0.963), as shown in Table 4.

**Table 4:** Comparison of failure modes before PSM

failure modes	LD	HD	χ^2^	*P*
No	37(36.6)	85(42.9)	1.097	0.295
Local recurrence	25(24.8)	36(18.2)	1.778	0.182
Distant metastasis	39(38.6)	77(38.9)	0.002	0.963

Further analysis showed that 18 cases (17.8%) of local recurrence in the LD group had esophageal recurrence and 7 cases (6.9%) had regional lymph node recurrence. Of the distant metastases, 16 (15.8%) had liver metastases, 8 (7.9%) had distant lymph node metastases, 5 (5.0%) had bone metastases, 2 (2.0%) had brain metastases, 4 (4.0%) had lung metastases, 2 (2.0%) had adrenal metastases, 1 (1.0%) had abdominal metastases, and 1 (1.0%) had pleural metastases. In the HD group, there were 23 cases (11.6%) of esophageal recurrence and 13 cases (6.6%) of regional lymph node recurrence. Of the distant metastases, 26 (13.1%) had liver metastases, 13 (6.6%) had distant lymph node metastases, 7 (3.5%) had bone metastases, 3 (1.5%) had brain metastases, 23 (11.6%) had lung metastases, and 5 (2.5%) had adrenal metastases.

### After PSM

#### Univariate and multivariate analysis and comparison of local control rates after PSM in the two groups

**Fig. 4:**
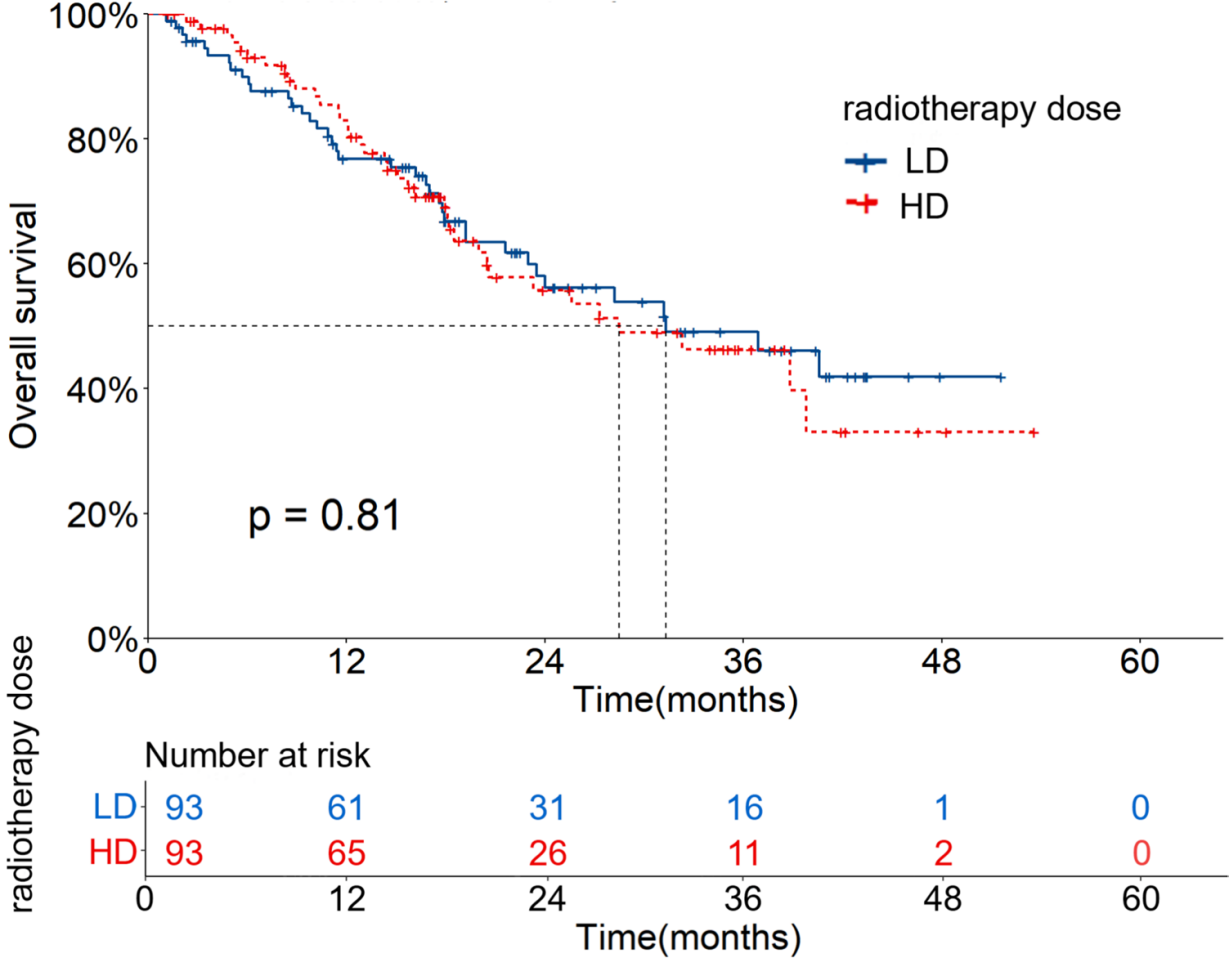
OS curves of patients with different radiotherapy doses after PSM

**Fig. 5:**
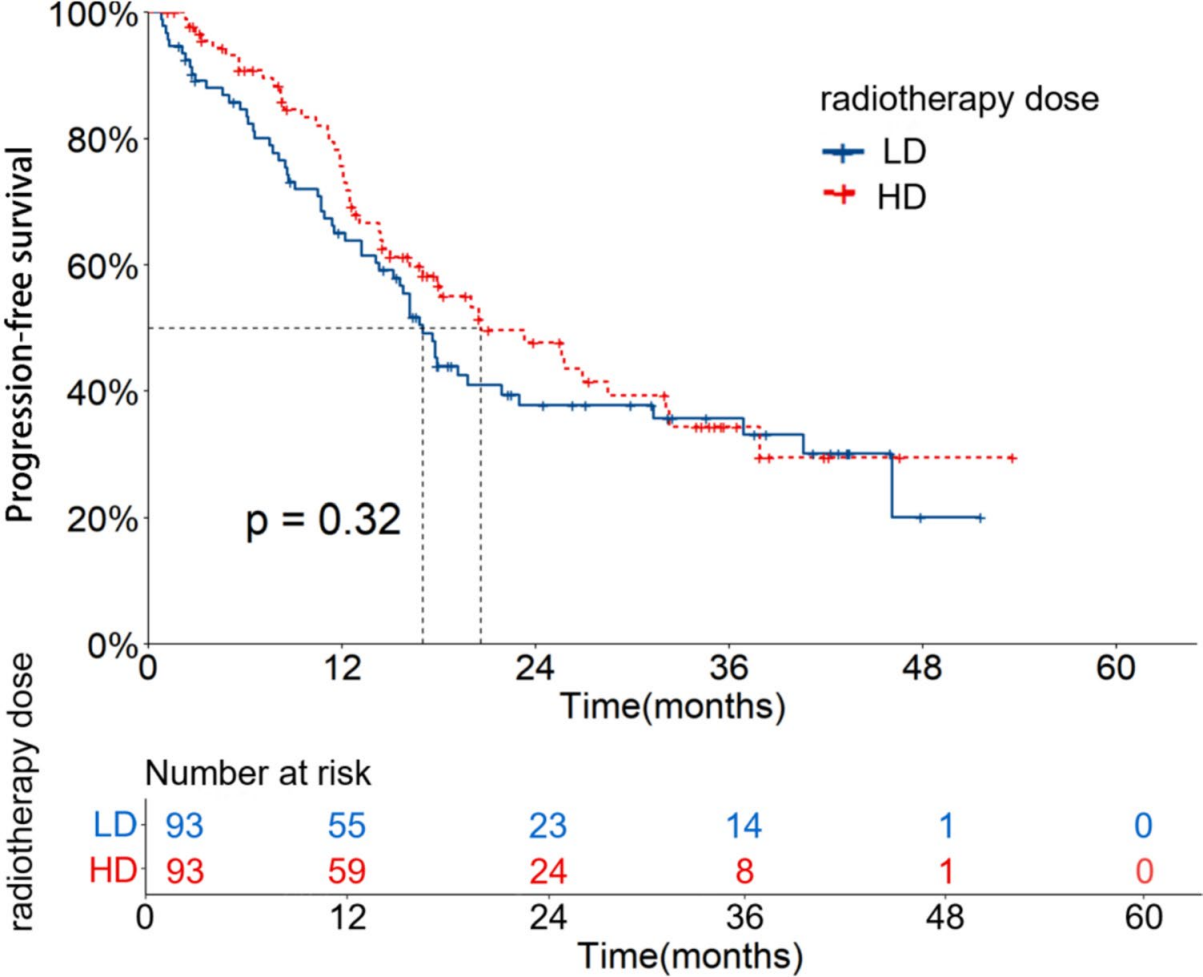
PFS curves of patients with different radiotherapy doses after PSM

The median follow-up time of 186 patients after PSM was 25.5 (95%CI: 18.6–32.4) months. The OS and PFS rates at 1, 2 and 3 years were 79.8%, 56.0%, 47.8% and 70.2%, 42.4%, 35.2%, respectively. The mOS and mPFS were 31.3 (95%CI: 20.5–42.2) and 17.9 (95%CI: 14.1–21.8) months, respectively. In the LD group, the OS and PFS rates at 1, 2 and 3 years were 76.8%, 56.2%, 49.1% and 65.0%, 37.8%, 35.7%, respectively. The mOS and mPFS were 31.3 (95%CI: 16.5–46.2) and 17.0 (95%CI: 15.1–19.0) months, respectively. In the HD group, the OS and PFS rates at 1, 2 and 3 years were 82.9%, 55.8%, 46.3% and 75.7%, 47.7%, 34.4%, respectively. The mOS and mPFS were 28.5 (95%CI: 16.1–40.9) and 20.6 (95%CI: 13.5–27.8) months, respectively. There were no significant differences between the two groups (X^2^ = 0.057, 0.974, *P* = 0.811, 0.324). The OS and PFS curves are shown in Figs. 4 and 5.

According to univariate analysis, age, gender, ECOG PS score and number of immunotherapy cycles were significant factors affecting OS (HR = 1.79, 1.64, 2.41, 0.55, *P* = 0.045, 0.034, 0.002, 0.015). ECOG PS score and tumor site were significant factors affecting PFS (HR = 2.36, 0.41, *P* = 0.002, 0.036). According to Cox multivariate analysis, patients < 70 years old, ECOG PS score 0–1 and number of immunotherapy cycles ≥ 3 had better OS (HR = 1.70, 2.48, 0.54, *P* = 0.026, 0.002, 0.014). Patients with ECOG PS scores 0–1 and upper thoracic segment had better PFS (HR = 2.36, 0.40, *P* = 0.002, 0.030). See Tables 2 and 3 for details.

In the LD group, 10 cases (10.8%) had PD, 25 cases (26.9%) had SD, 27 cases (29.0%) had PR, and 31 cases (33.3%) had CR. In the HD group, 9 cases (9.7%) had PD, 25 cases (26.9%) had SD, 27 cases (29.0%) had PR, and 32 cases (34.4%) had CR. The DCR values of LD and HD groups were 89.2% and 90.3%, respectively, with no statistical significance (X^2^ = 0.059, *P* = 0.809). The ORR values of LD and HD groups were 62.4% and 63.4%, respectively, with no statistical significance (X^2^ = 0.023, *P* = 0.879).

#### Subgroup analysis

Subgroup analysis of OS and PFS that may affect the two groups after matching.

**Fig. 6:**
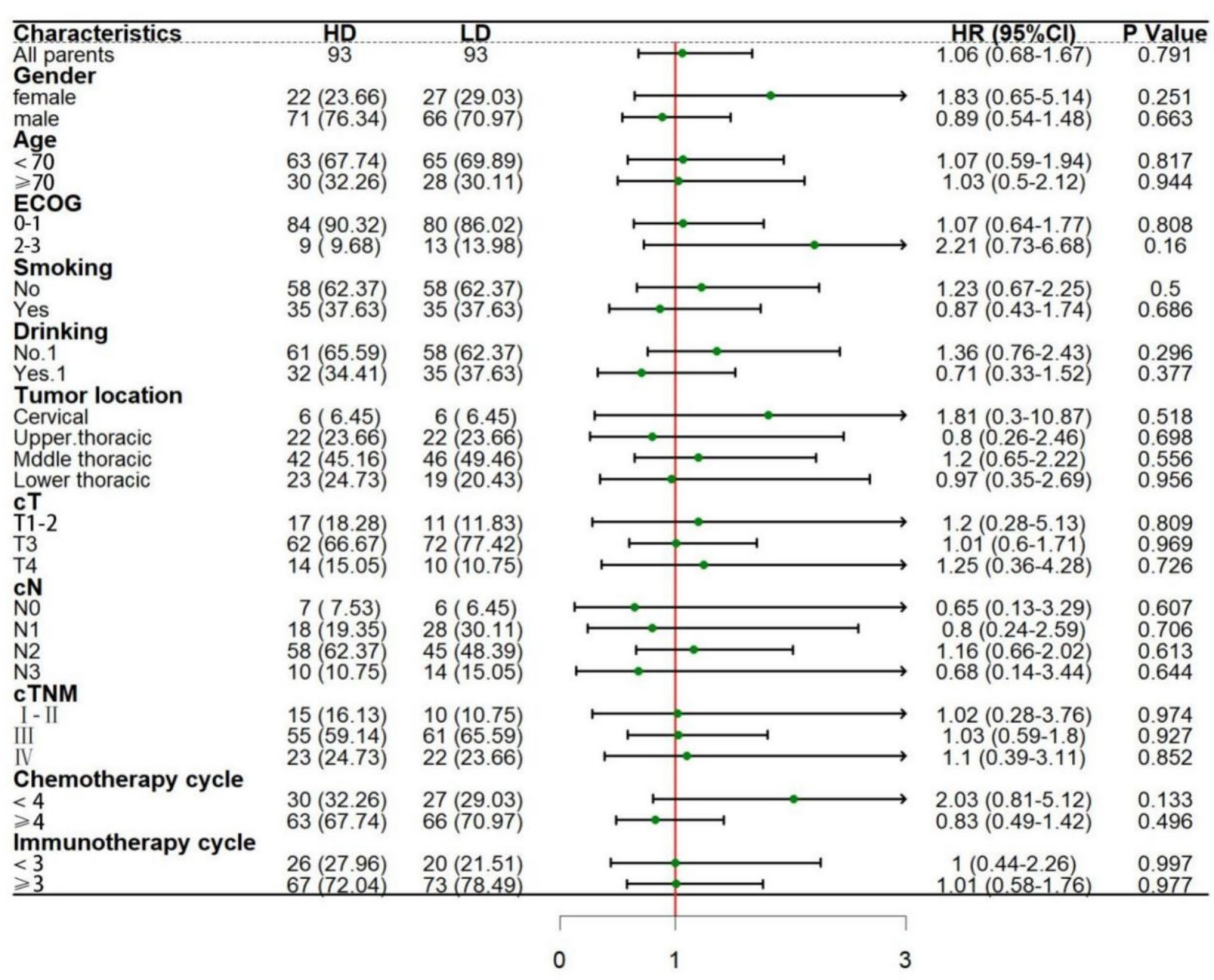
Subgroup analysis forest map of OS after PSM

**Fig. 7:**
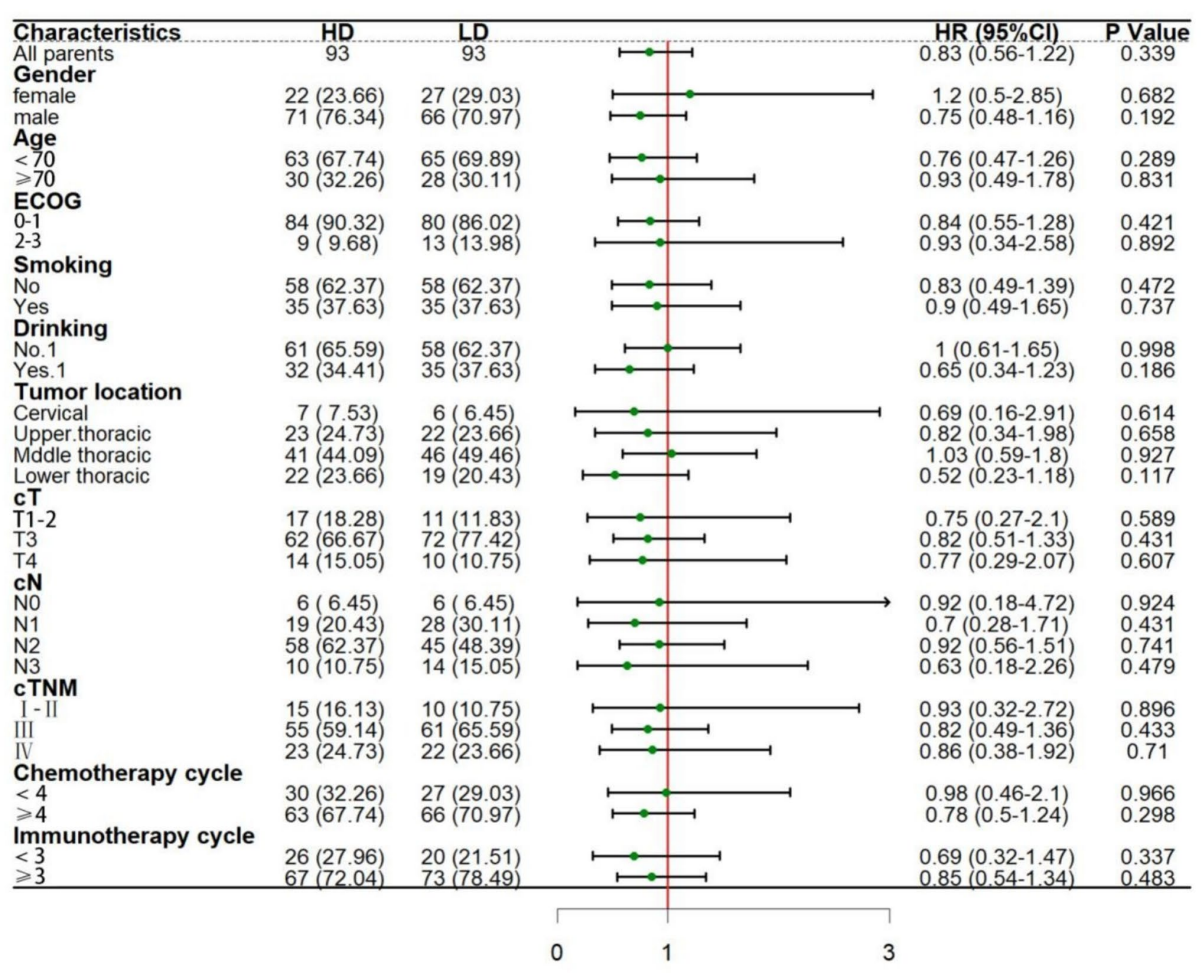
Subgroup analysis forest map of PFS after PSM

The results showed that ‌no significant difference was found in the current subgroup analysis. Additionally, there were no significant differences in OS (HR = 1.06, 95%CI: 0.68–1.67, *P* = 0.791) and PFS (HR = 0.83, 95%CI: 0.56–122, *P* = 0.339) between the two groups. This suggests that the efficacy of current treatment regimens in the whole population is not changed by risk stratification. However, attention should be paid to patients with ECOG score ≥ 2, number of chemotherapy cycles < 4, different lesion sites, and recommended cycles < 3. Moreover, it can be further verified by multi-dimensional biomarkers and expanded sample size in future studies (Figs. 6 and 7).

#### Failure mode and toxicity comparison

As of the follow-up date, 56 patients (60.2%) in the LD group had disease progression, of which 21 patients (22.6%) had local recurrence and 35 patients (37.6%) had distant metastasis. In the HD group, 46 cases (49.5%) had disease progression, of which 14 cases (15.1%) had local recurrence and 32 cases (34.4%) had distant metastasis, with no statistical significance (X^2^ = 2.171, 1.725, 0.210, *P* = 0.141, 0.189, 0.647), as shown in Table 5. Further analysis showed that 15 cases (16.1%) in the LD group had esophageal recurrence and 6 cases (6.5%) had regional lymph node recurrence. Of the distant metastases, 14 (15.1%) had liver metastases, 8 (8.6%) had distant lymph node metastases, 4 (4.3%) had bone metastases, 2 (2.2%) had brain metastases, 4 (4.3%) had lung metastases, 2 (2.2%) had adrenal metastases, and 1 (1.1%) had abdominal wall metastases. In the HD group, 10 patients (10.8%) had esophageal recurrence and 4 patients (4.3%) had regional lymph node recurrence. Of the distant metastases, 11 (11.8%) had liver metastases, 6 (6.5%) had distant lymph node metastases, 3 (3.2%) had bone metastases, 1 (1.1%) had brain metastases, 8 (8.6%) had lung metastases, and 3 (3.2%) had adrenal metastases.

**Table 5:** Comparison of failure modes after PSM

failure modes	LD	HD	χ^2^	*P*
No	37(39.8)	47(50.5)	2.171	0.141
Local recurrence	21(22.6)	14(15.1)	1.725	0.189
Distant metastasis	35(37.6)	32(34.4)	0.210	0.647

Pearson χ^2^ test

The incidence of treatment-related toxicity was 53 cases (57.0%) in the LD group and 44 cases (47.3%) in the HD group. The results showed that the toxicity trend of HD was lower but not significant, and the difference was not statistically significant (X^2^ = 1.745, *P* = 0.186). The adverse reactions in this study mainly included myelosuppression, radiotherapy-related side effects, immunotherapy-related side effects, and esophageal fistula. There was no statistically significant difference between the two groups. Among them, radiotherapy-related adverse reactions included radiation pneumonitis, radiation esophagitis, and radiation dermatitis. There were 14 cases of radiation esophagitis in the LD group and 16 cases in the HD group. There was no statistically significant difference between the two groups (X^2^ = 1.240, *P* = 0.265). There were 6 cases of radiation dermatitis in the LD group and 2 cases in the HD group. There was no statistically significant difference between the two groups (X^2^ = 1.176, *P* = 0.278). There were 4 cases of radiation pneumonitis in the LD group and 7 cases in the HD group. There was no statistically significant difference between the two groups (X^2^ = 0.870, *P* = 0.351). Adverse reactions related to immunotherapy included reactive capillary hyperplasia, pneumonia, hypothyroidism, myocarditis, and hypophysitis. There were 3 cases of immune hypothyroidism in the LD group and 0 cases in the HD group, and the difference was not statistically significant(X^2^ = 1.355, *P* = 0.244). There were 3 cases of immune-reactive capillary hyperplasia in the LD group and 0 cases in the HD group, and the difference was not statistically significant(X^2^ = 1.355, *P* = 0.244). There were 0 cases of immune myocarditis in the LD group and 3 cases in the HD group, and the difference was not statistically significant(X^2^ = 1.355, *P* = 0.244). There were 0 cases of immune pneumonia in the LD group and 3 cases in the HD group. The difference was not statistically significant(X^2^ = 1.355, *P* = 0.244). There were 0 cases of immune hypophysitis in the LD group and 1 case in the HD group, and the difference was not statistically significant(X^2^ = 0.000, *P* = 1.000). See Table 6 for details.

**Table 6:** Adverse reactions after PSM

Toxic and side reaction	LD (N = 93)						HD (N = 93)						χ^2^	*P*
	0	1	2	3	4	5	0	1	2	3	4	5		
No	40(43.0)						49(52.7)						1.745^a^	0.186^a^
Myelosuppression	0	8(8.6)	13(14.0)	10(10.8)	2(2.2)	–	0	7(7.5)	10(10.8)	8(8.6)	2(2.2)	–	1.216^a^	0.270^a^
Radiation esophagitis	0	7(7.5)	5(5.4)	2(2.2)	0	–	0	6(6.5)	7(7.5)	3(3.2)	0	–	1.240^a^	0.265^a^
Radiation dermatitis	0	5(5.4)	1(1.1)	0	0	–	0	2(2.2)	0	0	0	–	1.176	0.278
Radiation pneumonia	0	3(3.2)	1(1.1)	0	0	–	0	4(4.3)	3(3.2)	0	0	–	0.870^a^	0.351^a^
Esophageal fistula	–	0	0	2(2.2)	–	–	–	0	0	1(1.1)	–	–	0.356	0.550
Immune hypothyroidism	–	2(2.2)	1(1.1)	0	0	–	–	0	0	0	0	–	1.355	0.244
Immune capillary hyperplasia	–	1(1.1)	2(2.2)	0	0	0	–	0	0	0	0	0	1.355	0.244
Immune-associated myocarditis	–	0	0	0	0	–	–	0	1(1.1)	2(2.2)	0	–	1.355	0.244
Immune pneumonia	–	2(2.2)	0	0	0	–	–	2(2.2)	0	1(1.1)	0	–	0.000	1.000
Immune hypophysitis	–	0	0	0	0	–	–	1(1.1)	0	0	0	–	0.000	1.000

^a^was Pearson χ^2^ test, and the rest were continuously corrected χ^2^ test

## Discussion

In this study, multi-center real-world data from China were retrospectively analyzed to investigate the effect of radiotherapy dose (50.4 Gy vs. 60 Gy) on the prognosis of ESCC patients during the triple therapy. There were no significant differences in PFS, OS, local control rate, or toxicity between the two groups. This finding challenges the conventional notion of radiotherapy dose optimization, which suggests that radiotherapy doses may not be a key prognostic factor in the era of immunotherapy.

In traditional radical chemoradiotherapy, the radiotherapy dose is closely related to the local control rate [27]. However, the addition of immunotherapy may amplify the “distal effect” of radiotherapy by changing the tumor microenvironment (TME) and activating systemic anti-tumor immune responses [28–30]. Thus, radiotherapy may enhance systemic anti-tumor immunity through the release of tumor antigens in synergy with immunotherapy, which may partially offset differences in local control due to dose differences. In this study, there were no significant differences in local control rates between the LD and HD groups (89.2% vs. 90.3%). This could be attributed to the systemic regulatory effects of immunotherapy, making low-dose radiotherapy sufficient to trigger effective immune activation [31, 32]. The SCIENCE study [33] indicated that the pathological complete response rate (pCR) of esophageal cancer was as high as 60% in the neoadjuvant chemoradiotherapy group (41.4 Gy). The figure was significantly higher than that in the chemoradiotherapy group (47.3%), which suggested that low-dose radiotherapy combined with immunotherapy may achieve efficient tumor killing through immune cooperation. Therefore, the synergistic effect of immunotherapy may, to some extent, weaken the dependence on radiotherapy doses in patients receiving triple therapy for esophageal cancer.

Subgroup analysis showed that patients < 70 years old with ECOG score 0–1, and immunotherapy cycle number ≥ 3 had better OS, while patients with ECOG score 0–1 and upper thoracic tumors had better PFS. This suggests that patient baseline characteristics, such as physical condition, may influence prognosis more than radiotherapy dose. Therefore, individualized therapy should be combined with the exploration of individualized biomarkers. Based on the analysis data of the JUPITER 06 study [3], Chen’s research team [34] established the genomic immunoecological classification (EGIC) of esophageal cancer, which broadened the biomarker exploration direction of the first-line “chemotherapy+PD-1 antibody” model of advanced ESCC. To provide a new means of immunotherapy decision-making for advanced ESCC, ESCC was divided into different subtypes of immunotherapy by genotype. It was proposed that the optimization of immunogenicity indicators (such as novel tumor mutation load) could more accurately predict the curative effect. In addition, Liu ZC’s team [35] demonstrated for the first time that SPRY1+PD1+CD8+T cells could be used as independent predictors of the clinical benefit of ESCC immunotherapy. This achievement fills the gap in the study of cellular and molecular mechanisms of esophageal cancer immunotherapy, which is of important clinical translational significance and prospect. It will also provide a new foundation and a new direction for exploring more accurate and effective ESCC immunotherapy strategies. Therefore, future studies need to integrate clinical features with molecular markers to identify patients who benefit from low-dose radiotherapy, rather than relying solely on dose adjustment.

Additionally, the balance between radiotherapy dose and the toxic response of esophageal cancer combined with immunochemoradiotherapy is the focus and difficulty in current research. In this study, the incidence of treatment-related adverse events (TRAE) in the LD group (57.0%) was slightly higher than that in the HD group (47.3%), but the difference was not statistically significant. At present, multiple stages I/II single-arm small-sample clinical studies have been conducted for treating patients with unresectable locally advanced esophageal cancer with combined immunotherapy. Zhang et al. [14] used carrilizumab combined with concurrent chemoradiotherapy to treat 20 patients with locally advanced ESCC at a radiotherapy dose of 60 Gy. The results showed that the 24-month OS and PFS rates were 69.6% and 65.0%, respectively. The incidence of adverse reactions was 45% for grade $\geq$ treatment, 20% for grade $\geq$ radiation esophagitis, 10% for grade $\geq$ esophageal fistula, and zero for grade $\geq$ radiation pneumonia. In addition, Wang et al. [36] used untreated and unresectable stage $\mathrm{I}\!\mathrm{I}\!$ ~ $\mathrm{I}\!\mathrm{V}\!$A ESCC with a combination of triplizumab and radical chemoradiotherapy to treat 42 patients at a radiotherapy dose of 50.4 Gy/28 times. The results showed that the 3-year PFS and OS rates were 35.7% (95%CI: 23.8–53.6) and 44.8% (95%CI: 31.9–62.8), respectively. Also, 67% of patients (28/42) developed grade 1–2 immune-related adverse event (irAE) and 2% (1/42) developed grade 3 irAE. No life-threatening irAE occurred. As far as the current evidence is concerned, single-arm small-sample phase $\mathrm{I}\!$/$\mathrm{I}\!\mathrm{I}\!$ clinical trials have shown that immune concurrent chemoradiotherapy further improves the efficacy compared with traditional chemoradiotherapy in the treatment of locally advanced esophageal cancer, with controllable adverse reactions and good tolerance. The current consensus is that 50–54 Gy combined immunotherapy is the optimal range for balancing efficacy and safety. In clinical practice, the dose should be dynamically adjusted according to the patient’s baseline status (such as age, and comorbidity), molecular characteristics (PD-L1, ctDNA), and real-time toxic reactions. Future phase III clinical trials, such as RATIONALE 311 [37] and KEYNOTE-975 [38], should be used to further validate the individualized dosing strategy.

The findings indicate that a dose escalation of 60 Gy may not be necessary for ESCC patients receiving triple therapy. Low-dose radiotherapy (50.4 Gy) can be prioritized in clinical practice to reduce potential toxicity while incorporating individual patient characteristics and biomarkers to develop protocols. Moreover, attention should be paid to the positive impact of the number of immunotherapy cycles (≥ 3 cycles) on survival. This is consistent with the trend of the long-term benefit of tiriplizumab combined with chemotherapy in the JUPITER-06 study [3].

There are several limitations in the multicenter and retrospective study. The retrospective design may lead to selection bias, and a small sample size (especially the ECOG ≥ 2 subgroup) may mask potential differences. Most radiotherapy regimens are reviewed in different sub-centers, and there is a lack of unified multi-center review and verification. Differences in radiotherapy techniques may also affect radiotherapy dose and treatment outcome. What’s more, there is also a lack of biomarker analysis. It is mainly targeted at ESCC, which may not be suitable for the esophageal adenocarcinoma population. At present, this study includes 7 domestic institutions, but lacks the participation of representative institutions from key regions such as Beijing and Shanghai, which may affect the universality of this study. Therefore, prospective randomized controlled studies from multi-center institutions covering key areas are still needed to explore the best model of triple therapy, compare the survival differences of different doses combined with immunotherapy, and determine how immunotherapy alters the dose–response relationship of radiotherapy.

In conclusion, increasing the radiotherapy dose from 50.4 Gy to 60 Gy failed to achieve better survival and local control rates for patients with advanced ESCC receiving chemoradiotherapy combined with immunotherapy as the first-line therapy. Therefore, the traditional role of radiotherapy dose requires re-examination in the context of immunotherapy reshaping the treatment pattern of esophageal cancer. This study provides real-world evidence for the “non-critical hypothesis of radiotherapy dose” and requires further validation through prospective studies. In the future, individualized precision therapy strategies should focus more on immune microenvironment regulation, biomarker screening and multidisciplinary collaboration, rather than limited to dose optimization.

## Conclusion

This study concluded that for patients with advanced ESCC receiving chemoradiotherapy combined with immunotherapy as the first-line therapy, increasing the radiotherapy dose from 50.4 Gy to 60 Gy did not produce better survival and local control rates, nor did adverse reactions increase. More large-scale prospective studies may be needed for further verification in the future.

## Data Availability

The data that support the findings of this study are available from the corresponding author, [author initials], upon reasonable request.

## References

[1] Bray F, Laversanne M, Sung H, Ferlay J, Siegel RL, Soerjomataram I et al (2024) Global cancer statistics 2022: globocan estimates of incidence and mortality worldwide for 36 cancers in 185 countries. CA Cancer J Clin 74(3):229–263. 10.3322/caac.21834 38572751 10.3322/caac.21834

[2] Shah MA, Altorki N, Patel P, Harrison S, Bass A, Abrams JA (2023) Improving outcomes in patients with oesophageal cancer. Nat Rev Clin Oncol 20(6):390–407. 10.1038/s41571-023-00757-y 37085570 10.1038/s41571-023-00757-y

[3] Wang ZX, Cui C, Yao J, Zhang Y, Li M, Feng J et al (2022) Toripalimab Plus Chemotherapy in Treatment-Naïve, Advanced Esophageal Squamous Cell Carcinoma (Jupiter-06): A Multi-Center Phase 3 Trial. Cancer Cell 40(3):277–288. 10.1016/j.ccell.2022.02.007 35245446 10.1016/j.ccell.2022.02.007

[4] Doki Y, Ajani JA, Kato K, Xu J, Wyrwicz L, Motoyama S et al (2022) Nivolumab combination therapy in advanced esophageal squamous-cell carcinoma. N Engl J Med 386(5):449–462. 10.1056/NEJMoa2111380 35108470 10.1056/NEJMoa2111380

[5] Xu J, Kato K, Raymond E, Hubner RA, Shu Y, Pan Y et al (2023) Tislelizumab plus chemotherapy versus placebo plus chemotherapy as first-line treatment for advanced or metastatic oesophageal squamous cell carcinoma (Rationale-306): a global, randomised, placebo-controlled, phase 3 study. Lancet Oncol 24(5):483–495. 10.1016/s1470-2045(23)00108-0 37080222 10.1016/S1470-2045(23)00108-0

[6] Xin Z, Liu Q, Ai D, Chen K, Mariamidze E, Sumon MA et al (2023) Radiotherapy for advanced esophageal cancer: from palliation to curation. Curr Treat Options Oncol 24(11):1568–1579. 10.1007/s11864-023-01134-8 37812321 10.1007/s11864-023-01134-8

[7] Demaria S, Golden EB, Formenti SC (2015) Role of local radiation therapy in cancer immunotherapy. JAMA Oncol 1(9):1325–1332. 10.1001/jamaoncol.2015.2756 26270858 10.1001/jamaoncol.2015.2756

[8] Demaria S, Coleman CN, Formenti SC (2016) Radiotherapy: changing the game in immunotherapy. Trends Cancer 2(6):286–294. 10.1016/j.trecan.2016.05.002 27774519 10.1016/j.trecan.2016.05.002 PMC5070800

[9] Tian L, Goldstein A, Wang H, Ching Lo H, Sun Kim I, Welte T et al (2017) Mutual regulation of tumour vessel normalization and immunostimulatory reprogramming. Nature 544(7649):250–254. 10.1038/nature21724 28371798 10.1038/nature21724 PMC5788037

[10] Ai D, Hao S, Shen W, Wu Q, Zhang S, Chen Y et al (2024) Induction sintilimab and chemotherapy followed by concurrent chemoradiotherapy for locally advanced esophageal cancer: a proof-of-concept, single-arm, multicenter, phase 2 trial. EClinicalMedicine 69:102471. 10.1016/j.eclinm.2024.102471 38356729 10.1016/j.eclinm.2024.102471 PMC10864194

[11] Sharabi AB, Lim M, DeWeese TL, Drake CG (2015) Radiation and checkpoint blockade immunotherapy: radiosensitisation and potential mechanisms of synergy. Lancet Oncol 16(13):e498-509. 10.1016/s1470-2045(15)00007-8 26433823 10.1016/S1470-2045(15)00007-8

[12] Zhang W, Yan C, Gao X, Li X, Cao F, Zhao G et al (2021) Safety and feasibility of radiotherapy plus camrelizumab for locally advanced esophageal squamous cell carcinoma. Oncologist 26(7):e1110–e1124. 10.1002/onco.13797 33893689 10.1002/onco.13797 PMC8265339

[13] Zhu Y, Wen J, Li Q, Chen B, Zhao L, Liu S et al (2023) Toripalimab combined with definitive chemoradiotherapy in locally advanced oesophageal squamous cell carcinoma (Ec-Crt-001): a single-arm, phase 2 trial. Lancet Oncol 24(4):371–382. 10.1016/s1470-2045(23)00060-8 36990609 10.1016/S1470-2045(23)00060-8

[14] Zhang W, Yan C, Zhang T, Chen X, Dong J, Zhao J et al (2021) Addition of camrelizumab to docetaxel, cisplatin, and radiation therapy in patients with locally advanced esophageal squamous cell carcinoma: a phase 1b study. Oncoimmunology 10(1):1971418. 10.1080/2162402x.2021.1971418 34616588 10.1080/2162402X.2021.1971418 PMC8489938

[15] Li J, Wang X, Cao J, Fan C, Xiao Q, Zheng Z et al (2024) Immunochemotherapy Plus Radiotherapy Versus Immunochemotherapy Alone as First-Line Treatment for Treatment-Naive, Advanced Esophageal Squamous Cell Carcinoma (Aec-Icr-1st): A Multi-Center Cohort Study. Cancer Lett. 10.1016/j.canlet.2024.217411 39736452 10.1016/j.canlet.2024.217411

[16] Cooper JS, Guo MD, Herskovic A, Macdonald JS, Martenson JA Jr., Al-Sarraf M et al (1999) Chemoradiotherapy of locally advanced esophageal cancer: long-term follow-up of a prospective randomized trial (RTOG 85-01). Radiation therapy oncology group. JAMA 281(17):1623–1627. 10.1001/jama.281.17.1623 10235156 10.1001/jama.281.17.1623

[17] Kumar S, Dimri K, Khurana R, Rastogi N, Das KJ, Lal P (2007) A randomised trial of radiotherapy compared with cisplatin chemo-radiotherapy in patients with unresectable squamous cell cancer of the esophagus. Radiother Oncol 83(2):139–147. 10.1016/j.radonc.2007.03.013 17445928 10.1016/j.radonc.2007.03.013

[18] Minsky BD, Pajak TF, Ginsberg RJ, Pisansky TM, Martenson J, Komaki R et al (2002) Int 0123 (radiation therapy oncology group 94–05) phase Iii trial of combined-modality therapy for esophageal cancer: high-dose versus standard-dose radiation therapy. J Clin Oncol 20(5):1167–1174. 10.1200/jco.2002.20.5.1167 11870157 10.1200/JCO.2002.20.5.1167

[19] Welsh J, Settle SH, Amini A, Xiao L, Suzuki A, Hayashi Y et al (2012) Failure patterns in patients with esophageal cancer treated with definitive chemoradiation. Cancer 118(10):2632–2640. 10.1002/cncr.26586 22565611 10.1002/cncr.26586 PMC3747650

[20] Machtay M, Bae K, Movsas B, Paulus R, Gore EM, Komaki R et al (2012) Higher biologically effective dose of radiotherapy is associated with improved outcomes for locally advanced non-small cell lung carcinoma treated with chemoradiation: an analysis of the Radiation Therapy Oncology Group. Int J Radiat Oncol Biol Phys 82(1):425–434. 10.1016/j.ijrobp.2010.09.004 20980108 10.1016/j.ijrobp.2010.09.004 PMC5764542

[21] Yamoah K, Showalter TN, Ohri N (2015) Radiation therapy intensification for solid tumors: a systematic review of randomized trials. Int J Radiat Oncol Biol Phys 93(4):737–745. 10.1016/j.ijrobp.2015.07.2284 26530740 10.1016/j.ijrobp.2015.07.2284 PMC4635974

[22] Hulshof M, Geijsen ED, Rozema T, Oppedijk V, Buijsen J, Neelis KJ et al (2021) Randomized study on dose escalation in definitive chemoradiation for patients with locally advanced esophageal cancer (Artdeco Study). J Clin Oncol 39(25):2816–2824. 10.1200/JCO.20.03697 34101496 10.1200/JCO.20.03697

[23] Song T, Liang X, Fang M, Wu S (2015) High-dose versus conventional-dose irradiation in cisplatin-based definitive concurrent chemoradiotherapy for esophageal cancer: a systematic review and pooled analysis. Expert Rev Anticancer Ther 15(10):1157–1169. 10.1586/14737140.2015.1074041 26235427 10.1586/14737140.2015.1074041

[24] Brower JV, Chen S, Bassetti MF, Yu M, Harari PM, Ritter MA et al (2016) Radiation dose escalation in esophageal cancer revisited: a contemporary analysis of the National Cancer Data Base, 2004 to 2012. Int J Radiat Oncol Biol Phys 96(5):985–993. 10.1016/j.ijrobp.2016.08.016 27869098 10.1016/j.ijrobp.2016.08.016

[25] Kim HJ, Suh YG, Lee YC, Lee SK, Shin SK, Cho BC et al (2017) Dose-response relationship between radiation dose and loco-regional control in patients with stage Ii-Iii esophageal cancer treated with definitive chemoradiotherapy. Cancer Res Treat 49(3):669–677. 10.4143/crt.2016.354 27737537 10.4143/crt.2016.354 PMC5512369

[26] Zhang W, Luo Y, Wang X, Han G, Wang P, Yuan W et al (2018) Dose-escalated radiotherapy improved survival for esophageal cancer patients with a clinical complete response after standard-dose radiotherapy with concurrent chemotherapy. Cancer Manag Res 10:2675–2682. 10.2147/cmar.S160909 30147366 10.2147/CMAR.S160909 PMC6097517

[27] Luo HS, Huang HC, Lin LX (2019) Effect of modern high-dose versus standard-dose radiation in definitive concurrent chemo-radiotherapy on outcome of esophageal squamous cell cancer: a meta-analysis. Radiat Oncol 14(1):178. 10.1186/s13014-019-1386-x 31623639 10.1186/s13014-019-1386-x PMC6798457

[28] Zhang YP, Guo ZQ, Cai XT, Rong ZX, Fang Y, Chen JQ et al (2025) Pai-1-driven Sfrp2(High) cancer-associated fibroblasts hijack the abscopal effect of radioimmunotherapy. Cancer Cell. 10.1016/j.ccell.2025.02.024 40086438 10.1016/j.ccell.2025.02.024

[29] Theelen W, Chen D, Verma V, Hobbs BP, Peulen HMU, Aerts J et al (2021) Pembrolizumab with or without radiotherapy for metastatic non-small-cell lung cancer: a pooled analysis of two randomised trials. Lancet Respir Med 9(5):467–475. 10.1016/s2213-2600(20)30391-x 33096027 10.1016/S2213-2600(20)30391-X

[30] Zhang Z, Liu X, Chen D, Yu J (2022) Radiotherapy combined with immunotherapy: the dawn of cancer treatment. Signal Transduct Target Ther 7(1):258. 10.1038/s41392-022-01102-y 35906199 10.1038/s41392-022-01102-y PMC9338328

[31] Wang H, Yao Z, Kang K, Zhou L, Xiu W, Sun J et al (2024) Preclinical Study and Phase Ii Trial of Adapting Low-Dose Radiotherapy to Immunotherapy in Small Cell Lung Cancer. Med 5(10):1237–1254. 10.1016/j.medj.2024.06.002 38964333 10.1016/j.medj.2024.06.002

[32] Herrera FG, Ronet C, de Ochoa Olza M, Barras D, Crespo I, Andreatta M et al (2022) Low-dose radiotherapy reverses tumor immune desertification and resistance to immunotherapy. Cancer Discov 12(1):108–133. 10.1158/2159-8290.Cd-21-0003 34479871 10.1158/2159-8290.CD-21-0003 PMC9401506

[33] Leng XF HW, Lyu JH,et al (2025) Preliminary results from the multicenter, randomized phase Iii trial (science):Comparing chemotherapy plus sintilimab and chemoradiotherapy plus sintilimab versus chemoradiotherapy for neoadjuvant treatment in resectable locally advanced esophageal squamous cell carcinoma.* ASCO GI* (2025) LBA329.

[34] Chen YX, Wang ZX, Jin Y, Zhao Q, Liu ZX, Zuo ZX et al (2023) An immunogenic and oncogenic feature-based classification for chemotherapy plus Pd-1 blockade in advanced esophageal squamous cell carcinoma. Cancer Cell 41(5):919–932. 10.1016/j.ccell.2023.03.016 37059106 10.1016/j.ccell.2023.03.016

[35] Liu Z, Zhang Y, Ma N, Yang Y, Ma Y, Wang F et al (2023) Progenitor-Like Exhausted Spry1(+)Cd8(+)t Cells Potentiate Responsiveness to Neoadjuvant Pd-1 Blockade in Esophageal Squamous Cell Carcinoma. Cancer Cell 41(11):1852–1870. 10.1016/j.ccell.2023.09.011 37832554 10.1016/j.ccell.2023.09.011

[36] Wang R, Ling Y, Chen B, Zhu Y, Hu Y, Liu M et al (2024) Long-term survival and post-hoc analysis of toripalimab plus definitive chemoradiotherapy for oesophageal squamous cell carcinoma: insights from the Ec-Crt-001 phase II trial. EClinicalMedicine 75:102806. 10.1016/j.eclinm.2024.102806 39281099 10.1016/j.eclinm.2024.102806 PMC11402426

[37] Yu R, Wang W, Li T, Li J, Zhao K, Wang W et al (2021) Rationale 311: tislelizumab plus concurrent chemoradiotherapy for localized esophageal squamous cell carcinoma. Future Oncol 17(31):4081–4089. 10.2217/fon-2021-0632 34269067 10.2217/fon-2021-0632

[38] Shah MA, Bennouna J, Doi T, Shen L, Kato K, Adenis A et al (2021) Keynote-975 study design: a phase III study of definitive chemoradiotherapy plus pembrolizumab in patients with esophageal carcinoma. Future Oncol 17(10):1143–1153. 10.2217/fon-2020-0969 33533655 10.2217/fon-2020-0969 PMC7927908

